# Lipid Metabolism as a Therapeutic Target in Arrhythmogenic Cardiomyopathy: Emerging Therapeutic Implications of Lipid-Lowering Therapy

**DOI:** 10.31083/RCM51364

**Published:** 2026-08-21

**Authors:** Nicolò Pasini, Federico Barocelli, Alberto Bettella, Alessia Ristagno, Mattia Santoro, Eleonora Canu, Antonio Crocamo, Filippo Luca Gurgoglione, Laura Torlai Triglia, Francesca Russo, Angela Guidorossi, Nicola Gaibazzi, Maria Francesca Notarangelo, Gian Luca Gonzi, Giampaolo Niccoli

**Affiliations:** ^1^Cardiology Division, Parma University Hospital, 43126 Parma, Italy; ^2^Cardiology Division, University of L’Aquila, 67100 L’Aquila, Italy

**Keywords:** arrhythmogenic cardiomyopathy, inflammation, cholesterol, statin

## Abstract

Arrhythmogenic cardiomyopathy (ACM) is a hereditary myocardial disorder characterized by progressive replacement of ventricular myocardium with fibro-fatty tissue, leading to life-threatening ventricular arrhythmias and heart failure. Historically, management has focused on arrhythmia suppression and prevention of sudden cardiac death (SCD). However, recent advances in understanding disease mechanisms have redefined ACM as a structural–metabolic cardiomyopathy. Central to this evolving paradigm is the “adipogenic switch”, driven by desmosomal dysfunction, impaired Wnt/β-catenin signaling, and activation of the peroxisome proliferator-activated receptor gamma (PPARγ) pathway. Emerging evidence highlights the critical role of metabolic dysregulation, specifically the oxidized low-density lipoprotein (oxLDL)/cluster of differentiation 36 (CD36)/PPARγ axis, in driving disease progression. Elevated oxLDL levels correlate with greater phenotypic severity, increased ventricular fat infiltration, and a higher arrhythmic risk. Translational models demonstrate that high-fat diets exacerbate the ACM phenotype, whereas lipid-lowering interventions, particularly statins, show potential to arrest fibro-fatty remodeling. In addition to the primary inhibition of 3-hydroxy-3-methylglutaryl-coenzyme A (HMG-CoA) reductase associated with statins, these medications exert pleiotropic effects, including attenuation of systemic inflammation, stabilization of the intercalated disc via Rho GTPase modulation, and reduction of oxidative stress. Clinical data from non-ischemic dilated cardiomyopathy (NIDCM) suggest that lipophilic statins such as atorvastatin, may confer significant mortality and antiarrhythmic benefits by stabilizing the myocardial electrical substrate. The ongoing SEARCH trial (NCT06922994) represents a landmark effort to translate these insights into clinical practice. Using high-dose atorvastatin and sensitive primary endpoints, such as right ventricular free wall longitudinal strain, this study aimed to determine whether lipid-lowering therapy can function as a true disease-modifying intervention in ACM. The integration of lipid-lowering agents into the therapeutic armamentarium for ACM could enable a transition toward precision medicine, contingent upon successful clinical validation. If clinical trials confirm the efficacy of these agents in slowing myocardial substrate decay, this approach could redefine the management of ACM, including the use of targeted molecular strategies to preserve myocardial identity and electrical stability.

## 1. Introduction

Arrhythmogenic cardiomyopathy (ACM) is a hereditary myocardial disorder characterized by the progressive loss of ventricular myocytes and their replacement with fibro-fatty tissue. This process creates an arrhythmogenic substrate that predisposes to malignant arrhythmias [1,2,3]. Although historically described as an isolated disease of the right ventricle (arrhythmogenic right ventricular cardiomyopathy, ARVC), current clinical and genetic evidence redefines it as a disease capable of affecting both ventricles. This shift has led to the development of multiparametric diagnostic frameworks, such as the 2010 Task Force Criteria and the more recent Padua criteria [4,5,6,7]. Diagnosis relies on the integration of clinical and family history, electrocardiogram (ECG) abnormalities, multimodality imaging (specifically cardiac magnetic resonance, cMRI), and genetic testing alongside the emerging role of circulating biomarkers [1,8,9,10,11]. Therapeutic strategies focus on the prevention of sudden cardiac death (SCD), management of arrhythmic burden, and control of heart failure progression. Current interventions include lifestyle modification (restriction of high-intensity sports), β-blockers and other antiarrhythmic medications, as well as catheter ablation, heart failure pharmacotherapy, and the use of an implantable cardioverter-defibrillator (ICD) for SCD prevention. In this evolving landscape, characterized by precision diagnostics and targeted molecular therapies, lipid-lowering agents are undergoing a clinical reassessment [8,12,13,14]. While traditionally associated with the prevention of plaque-related injury, these agents have recently emerged as a focal point of research due to the hypothesized disease-modifying capacity of their pleiotropic effects within the specific context of ACM.

ACM demands focused clinical attention due to its substantial prevalence (1:1000 to 1:5000) [1] and disproportionate impact on young individuals, accounting for 11% of sudden cardiac deaths in young adults and 22% in athletes [1,15]. The disease is particularly devastating because SCD may be the first manifestation during a preclinical “concealed” phase, with 59% of sudden death victims having no prior cardiac symptoms [1,16]. Exercise not only triggers ventricular arrhythmias (72% of cardiac arrests occur during exertion) but also accelerates disease progression in desmosomal mutation carriers [17]. Diagnosis remains exceptionally challenging as the 2010 Task Force Criteria lack sensitivity for left ventricular involvement and early disease detection when electrical abnormalities precede structural changes, necessitating updated frameworks such as the Padua and proposed European Task Force criteria. Childhood-onset disease carries a particularly grave prognosis, with 23% experiencing severe cardiac events including heart transplantation [18]. These realities—high prevalence in young populations, asymptomatic sudden death, exercise-triggered events, diagnostic complexity, and severe paediatric outcomes [19,20]—underscore why emerging disease-modifying therapies, including lipid-lowering agents, warrant rigorous investigation.

## 2. Arrhythmogenic Cardiomyopathy as a Metabolic Disease: Revisiting Pathophysiology

### 2.1 Desmosomes Beyond Mechanics: Signaling and Cell Fate

Desmosomes are specialized intercellular junctions located at the intercalated discs that ensure mechanical integrity by anchoring intermediate filaments between adjacent cardiomyocytes [21]. They are composed of key proteins, including plakophilin-2 (PKP2), desmoplakin (DSP), desmoglein-2 (DSG2), desmocollin-2 (DSC2), and plakoglobin (JUP) [22]. While traditionally regarded as passive mechanical junctions, desmosomes are now recognized as dynamic signaling hubs that regulate pathways involved in cell differentiation, proliferation, and tissue homeostasis [23]. The classical “weak glue” hypothesis postulates that mutations in desmosomal genes reduce intercellular adhesion, rendering cardiomyocytes vulnerable to mechanical stress [24]. However, this purely mechanical explanation is insufficient to account for the progressive fibro-fatty myocardial remodeling that characterizes ACM [25]. Indeed, increasing evidence demonstrates that desmosomal dysfunction profoundly alters intracellular signaling pathways that govern cell fate and tissue remodeling [26].

### 2.2 The Adipogenic Switch and Myocardial Reprogramming

Adipogenesis in ACM is increasingly recognized as an active remodeling process rather than a passive consequence of myocyte loss [27]. According to the “differentiation theory”, pathogenic variants in desmosomal genes, such as those affecting *PKP2*, *DSP*, or *JUP*, disrupt not only mechanical coupling but also intracellular signaling, thereby diverting myocardial cell fate decisions [27].

A central mechanism involves the nuclear translocation of plakoglobin, which competes with β-catenin for binding to T cell factor (TCF)/lymphoid enhancer factor family (LEF) transcription factors, leading to the suppression of the canonical Wnt/β-catenin pathway [26]. Since Wnt signaling acts as a major inhibitor of adipogenesis, its repression enables the activation of the adipogenic transcriptional program through peroxisome proliferator-activated receptor gamma (PPARγ) and CCAAT-enhancer binding proteins (C/EBP) family members [27]. This signaling shift primarily affects resident cardiac mesenchymal stromal cells (C-MSCs) and fibro-adipogenic progenitors, which are redirected toward adipocyte differentiation rather than maintaining structural support [27].

Concomitantly, desmosomal remodeling activates the Hippo kinase cascade (mammalian ste20-like kinase 1/2 (MST1/2) and large tumor suppressor kinase 1/2 (LATS1/2)), likely through protein kinase C (PKC) inactivation and neurofibromatosis type 2/moesin-ezrin-radixin-like protein (NF2/Merlin) activation [23]. This results in the phosphorylation and cytoplasmic sequestration of YES-associated protein (YAP), which can physically interact with β-catenin, further reinforcing Wnt pathway suppression [23]. The coordinated suppression of Wnt/β-catenin and YAP/transcriptional coactivator with PDZ-binding motif (TAZ) signaling thus acts as a molecular switch driving progenitor cells toward adipocyte differentiation, resulting in the marked upregulation of PPARγ, the master regulator of adipogenesis [23,27]. Through these mechanisms, structural defects at the intercalated disc are directly translated into metabolic and phenotypic remodeling, redefining ACM as a disorder of altered myocardial identity rather than merely impaired mechanical or electrical conduction [27].

### 2.3 Oxidative Lipid Stress and the oxLDL/CD36/PPARγ Axis

Beyond genetic and structural abnormalities, ACM is increasingly recognized as a metabolic cardiomyopathy in which dysregulated lipid signaling plays a central pathogenic role [28]. Among these metabolic factors, oxidized low-density lipoprotein (oxLDL) has emerged as a critical pathogenic cofactor driving disease progression [29].

The interaction between oxLDL and lectin-like oxidized low-density lipoprotein receptor-1 (LOX-1) represents a central pathogenic mechanism in cardiovascular disease, orchestrating multiple downstream pathways including reactive oxygen species (ROS) generation, inflammatory signaling, desmosomal disruption, myocardial fibrosis, and fibro-fatty progression [30,31,32]. In cardiac tissue, oxLDL-LOX-1 signaling drives myocardial fibrosis through fibroblast proliferation and collagen deposition [33]. Additionally, oxLDL promotes fibro-fatty progression in arrhythmogenic cardiomyopathy through cluster of differentiation 36 (CD36)-mediated internalization and PPARγ-driven adipogenesis [29]. This multifaceted pathway creates a self-perpetuating cycle of oxidative stress, inflammation, barrier dysfunction, fibrosis, and adipogenic transformation that drives atherosclerosis progression and adverse cardiac remodeling.

Clinically, elevated circulating oxLDL levels strongly correlate with disease penetrance and severity: patients with overt ACM exhibit significantly higher plasma oxLDL concentrations than both healthy controls and mutation carriers without phenotypic expression [29]. Notably, patients with oxLDL levels exceeding a specific threshold (86 ng/mL) show a greater ventricular fat mass—typically progressing from the epicardium to the endocardium—and a higher prevalence of right ventricular and biventricular dysfunction [29]. Furthermore, increased oxLDL concentrations are independently associated with a greater long-term risk of major arrhythmic events [29]. Experimental models support this paradigm, as high-fat diets that increase oxLDL levels in Pkp2-mutant mice induce subepicardial adipogenesis.

At the molecular level, the pathogenic effects of oxLDL are largely mediated through activation of the oxLDL/CD36/PPARγ signaling axis [28]. Upon uptake via the CD36 scavenger receptor, oxLDL-derived components such as 13-hydroxyoctadecadienoic acid (13-HODE) directly induce PPARγ expression and activation [29]. A self-amplifying loop is thereby established, in which PPARγ upregulates CD36 expression, further promoting additional oxLDL uptake and accelerating the adipogenic differentiation of cardiac stromal cells [34].

Metabolomic studies in ACM reveal disease-specific alterations in polyunsaturated fatty acid (PUFA) composition driven by enzymatic remodeling of LDL particles [34]. Secretory phospholipase A2 (sPLA2) hydrolyses esterified lipids on the LDL surface, liberating polyunsaturated fatty acids such as linoleic and arachidonic acid, which are subsequently converted by 15-lipoxygenase (15-LO) into bioactive oxylipins, including 13-HODE [34]. Crucially, only the free forms of these oxidized fatty acids are capable of activating the oxLDL/CD36/PPARγ axis and inducing adipogenic differentiation of cardiac mesenchymal stromal cells [34].

In specific genetic contexts, such as *TMEM43 *p.Ser358Leu, a “heart–gut axis” has been described, whereby enhanced intestinal lipid absorption leads to systemic hyperlipidaemia and elevated plasma LDL levels, further exacerbating fibro-fatty myocardial remodeling [35].

These lipidomic signatures not only elucidate disease mechanisms but may also serve as promising biomarkers for early detection, phenotypic stratification, and therapeutic monitoring in ACM [29].

### 2.4 Inflammation, Autoimmunity, and Immune Dysregulation

Transcriptomic and proteomic profiling further reveal marked dysregulation of immune and inflammatory pathways in ACM hearts, including nuclear factor kappa-light-chain-enhancer of activated B cells (NF-κB) signaling, B-cell activation, and interferon responses [36]. Elevated levels of pro-inflammatory cytokines such as interleukin-1 (IL-1), interleukin-6 (IL-6), and tumour necrosis factor alpha (TNF-α) are thought to contribute to the severity of fibro-fatty remodeling [37]. Histologically, inflammatory infiltrates are present in over two-thirds of ACM hearts, and cardiotropic viruses have been detected in sporadic cases, suggesting that viral infection may act as an additional trigger contributing to disease onset and progression alongside the known genetic/dystrophic mechanisms involving desmosomal proteins [38,39]. Given the role of inflammation and potential infectious and immunological triggers, the clinical presentation of certain forms of ACM increasingly overlaps with episodes of myocarditis—referred to as “hot phases”—and can progress to severe, fulminant forms requiring heart transplantation [40,41]. ACM is also increasingly recognized as having an autoimmune component mediated by anti-desmosomal autoantibodies [25]. It is hypothesized that mechanical disruption of the intercalated disc—driven by genetic fragility or excessive exercise—exposes cryptic epitopes normally hidden from immune surveillance. This exposure triggers autoimmune responses that further destabilize cell–cell junctions and impair adaptive remodeling [25].

### 2.5 Exercise and Environmental Modulators of Disease Expression

The clinical expression of ACM reflects a threshold phenomenon in which genetic susceptibility and environmental stressors interact to determine disease onset, progression, and arrhythmic risk [42]. Individuals harbouring pathogenic desmosomal variants may develop the phenotype with minimal physical activity, whereas those with lower genetic risk require substantial environmental exposure, such as prolonged endurance exercise, to cross the phenotypic threshold [42]. Physical exercise represents the most potent environmental modifier in ACM, accelerating phenotypic expression and increasing the risk of life-threatening ventricular arrhythmias [24]. During intense physical activity, the increased hemodynamic load and repetitive mechanical traction overcome the reduced adhesive capacity of mutated desmosomes, leading to cardiomyocyte detachment, necrosis, and fibro-fatty replacement [43,44]. This vulnerability is especially pronounced in the right ventricle, where pulmonary vascular resistance decreases less than systemic resistance during exercise, resulting in disproportionately elevated wall stress [42].

Beyond its mechanical effects, exercise modulates key molecular pathways implicated in ACM progression. Endurance training is associated with the upregulation of PPARγ, perturbation of Wnt/β-catenin signaling, and the downregulation of essential intercalated disc genes such as *PKP2*, *DSG2*, and *DSC2*, thereby promoting adipogenic remodeling and progressive structural deterioration [25,45,46].

These observations have led to the formulation of the “desmosomal reserve theory”, which postulates that individuals with sufficient baseline desmosomal integrity can tolerate mechanical stress, whereas genetic predisposition or extreme cumulative exercise exhausts this reserve and precipitates disease [25].

In some cases, extreme endurance exercise alone may be sufficient to cause an “exercise-induced ACM” phenotype even in the absence of identifiable causal genetic variants—a subgroup often termed “gene-elusive”—supporting the notion that environmental stress can independently challenge intercalated disc integrity [25]. Consequently, the clinical trajectory of exercise-induced ACM may initiate with a phase of adaptive positive remodeling (the classic athlete’s heart), but eventually reaches a critical tipping point where the desmosomal reserve can no longer compensate for repetitive mechanical injury [25].

Metabolic status further modulates the phenotypic threshold. Elevated circulating oxLDL levels act as pathogenic triggers through the activation of the oxLDL/CD36/PPARγ axis, amplifying adipogenic differentiation and ventricular dysfunction, particularly in genetically susceptible individuals [28].

**Fig. 1.**
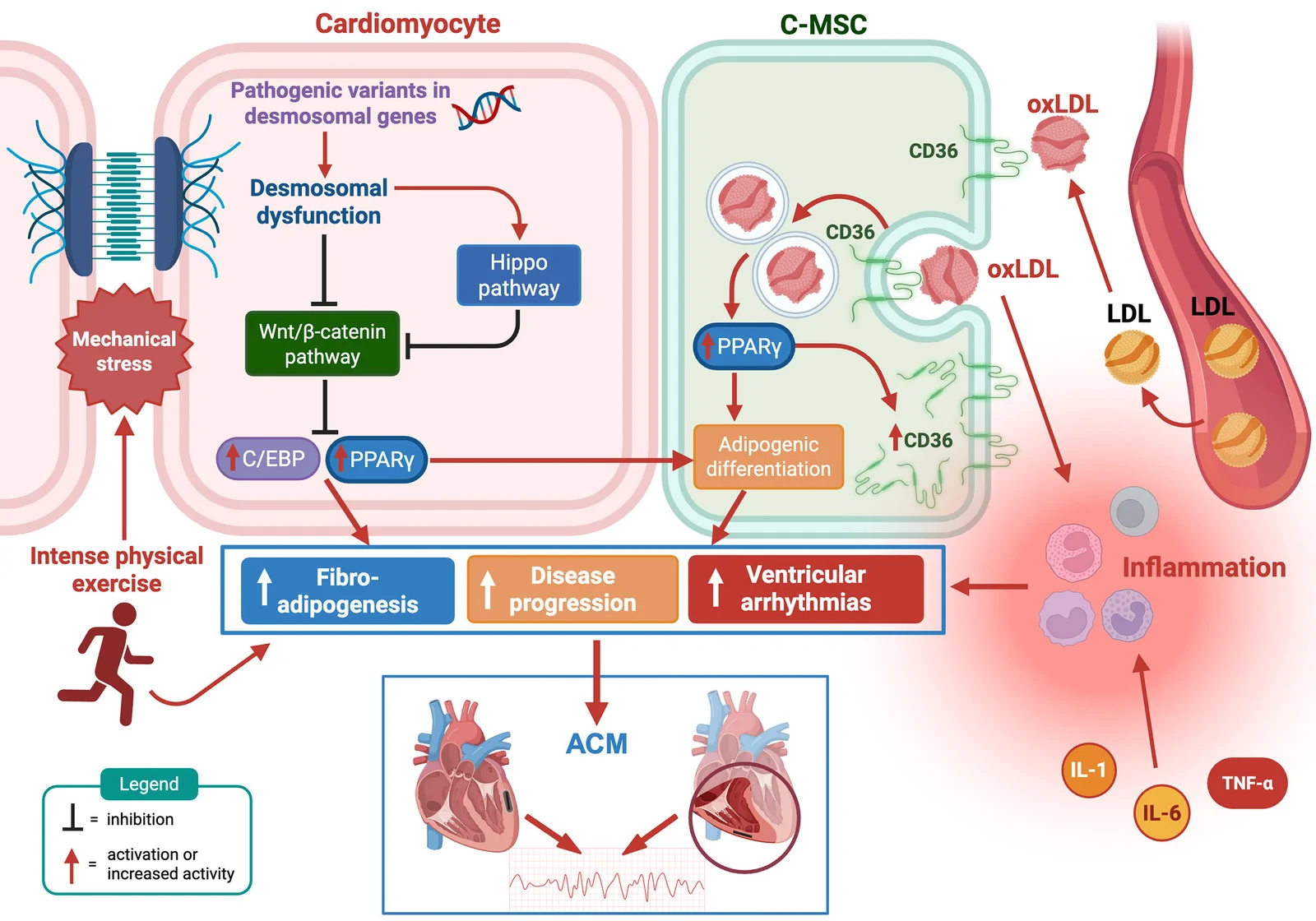
**Desmosomal dysfunction and molecular pathways in arrhythmogenic cardiomyopathy**. The figure provides an integrated schematic overview of the molecular mechanisms underlying arrhythmogenic cardiomyopathy. ACM, arrhythmogenic cardiomyopathy; CD36, cluster of differentiation 36; C/EBP, CCAAT/enhancer-binding protein; C-MSC, cardiac mesenchymal stromal cell; IL-1, interleukin-1; IL-6, interleukin-6; LDL, low-density lipoprotein; oxLDL, oxidized low-density lipoprotein; PPARγ, peroxisome proliferator-activated receptor gamma; TNF-α, tumour necrosis factor-alpha. This figure was created with BioRender.com.

Exercise also contributes to electrical instability by promoting gap junction remodeling, characterized by reduced Connexin-43 and Naᵥ1.5 expression at the intercalated disc, as well as by inducing abnormalities in calcium handling [47]. Clinically, athletes with ACM typically present with symptoms and arrhythmias approximately 10 years earlier than sedentary patients [24] and exhibit a five-fold increase in the risk of SCD [24]. Additional environmental stressors, including hypoxia, extreme temperatures, and psychosocial stress, may further exacerbate disease expression by increasing sympathetic drive and cardiac workload [21,47]. The altered molecular pathways are summarized in Fig. 1.

### 2.6 Integrated Pathogenic Model of Arrhythmogenic Cardiomyopathy

Collectively, ACM emerges as a multifactorial cardiomyopathy in which desmosomal dysfunction, altered cell fate signaling, metabolic derangement, inflammation, immune activation, and environmental stressors converge to determine disease penetrance, progression, and arrhythmic risk [25]. The transition from an electrically centric view to a structural–metabolic paradigm, together with the strong association between oxidative lipid burden and severe phenotypes, provides a unifying framework for understanding disease heterogeneity and opens new perspectives for targeted therapeutic strategies beyond conventional anti-arrhythmic approaches [21,28,29].

## 3. Translational Evidence Supporting Lipid-Lowering Strategies

As discussed in the previous chapters of this review, metabolic dysregulation appears to play a pivotal role in the molecular pathophysiology of ACM. Accordingly, in recent years, several research groups have focused on this aspect, generating translational evidence from both *in vitro* and *in vivo* models.

### 3.1 In Vitro Experiments

A central regulator of adipogenesis in ACM is PPARγ, whose activation may be triggered by the impairment of two major signaling pathways: Wnt/β-catenin [26] and Hippo [23].

Kim et al. [48] investigated the role of dysregulated PPARγ in induced pluripotent stem-cell–derived cardiomyocytes generated from the fibroblasts of a patient with clinically manifest ACM carrying a homozygous PKP2 mutation. Indomethacin and rosiglitazone, two known PPARγ activators, were added to the culture medium; after several weeks of treatment, the cells exhibited marked lipogenesis and increased apoptosis. As these compounds are not endogenous ligands, the authors subsequently sought an endogenous molecule capable of inducing aberrant PPARγ activation. This was identified as 13-HODE, a major component of oxLDL. In the same study, the authors also demonstrated that PPARγ hyperactivation requires preserved Peroxisome Proliferator-Activated Receptor alpha (PPARα) function to induce pathogenic cellular changes [48].

Sommariva et al. [29] further explored the role of the oxLDL/CD36/PPARγ axis in ACM pathogenesis. To test the hypothesis that oxLDL promotes adipogenesis in ACM cardiac mesenchymal stromal cells, ACM and healthy control (HC) C-MSCs were cultured in adipogenic medium (AM) supplemented with oxLDL. Exposure to oxLDL resulted in increased lipid accumulation in ACM C-MSCs compared with AM alone, along with the upregulation of CD36 and PPARγ expression. Based on the hypothesis that 13-HODE represents the key oxLDL component driving adipogenesis, the experiment was repeated using 13-HODE instead of oxLDL. Notably, 13-HODE significantly increased lipid accumulation exclusively in ACM C-MSCs, while no effect was observed in HC cells [29]. To directly assess the causal role of CD36 in the adipogenic process, CD36 was silenced using siRNA in ACM C-MSCs cultured in AM supplemented with oxLDL. A mean reduction in CD36 expression of approximately 32% was sufficient to significantly decrease lipid accumulation compared with non-silenced cells [29].

Chen and colleagues [49] performed a comparative proteomic analysis of explanted hearts from four patients with ACM and four with dilated cardiomyopathy (DCM), using non-diseased hearts as controls. They observed that, similarly to DCM cardiomyocytes, myocardial samples from ACM patients exhibited the downregulation of proteins involved in mitochondrial function, resulting in impaired cellular metabolic efficiency [49]. In contrast, inflammatory signaling pathways were significantly more activated in ACM than in DCM. Importantly, the authors identified a profound reprogramming of lipid metabolism in ACM, characterized by the upregulation of lipogenic factors such as fatty-acid binding protein-4 (FABP4) and fatty-acid synthase (FASN), with C/EBPα emerging as a key transcriptional regulator. PPARγ and C/EBPα were shown to reinforce each other through positive feedback loops [27], thereby sustaining high expression levels and promoting adipose tissue deposition.

A recent study by Takemoto and colleagues [50] elucidated the regulatory pathway involving TMEM43 and sterol regulatory element-binding proteins (SREBPs), which function as pivotal transcription factors in the control of adipogenesis and lipogenesis. The investigation demonstrates that TMEM43 serves as a negative regulator of SREBP activity by sequestering the transcriptional coactivator Leucine-rich PPR motif-containing protein (LRPPRC) at the nuclear membrane. Consequently, a deficiency in TMEM43 precipitates heightened SREBP signaling and accelerated adipocyte differentiation, offering a possible mechanistic explanation for the aberrant lipid accumulation observed in TMEM43-related ACM [50].

Impaired metabolic efficiency affects not only cardiomyocytes but also cardiac stromal cells (CStCs), which play a crucial role in adipose tissue accumulation in ACM [51]. Focusing on the epigenetic regulation of metabolic pathways, Volani et al. [52] investigated the role of the histone acetyltransferase general control non-depressible 5 (GCN5), an enzyme involved in adipogenesis and cardiac metabolic regulation, in CStCs. GCN5 expression was significantly increased in ACM samples compared with HC. Inhibition of GCN5 resulted in reduced lipid accumulation and the attenuation of reactive oxygen species production in ACM CStCs, supporting a reciprocal interplay between metabolic dysregulation and oxidative stress in promoting fibro-adipogenic remodeling [52]. Similarly, Vencato and colleagues [53] examined the role of histone deacetylases in cardiac fibro-adipogenic precursors in murine models of ACM. Administration of the pan–histone deacetylase inhibitor givinostat led to a reduction in the number of adipocytes generated under pro-adipogenic conditions. Consistently, RNA sequencing analysis revealed significant downregulation of key regulators of adipogenic differentiation and lipid metabolism, including PPARγ [53]. Epigenetic modulation of metabolic pathways may therefore synergize with lipid-lowering approaches in attenuating fibro-adipogenic remodeling in ACM.

### 3.2 In Vivo Experiments

Animal models of ACM, primarily rodents and zebrafish [54], have been extensively used to investigate disease onset and progression. More recently, several studies have focused on these models to explore the impact of impaired lipid metabolism on ACM development.

Centner et al. [55] employed a DSG2 knockout mouse model of ACM to evaluate whether a high-fat diet influenced disease progression compared with diseased mice on a standard diet and wild-type controls. Mice receiving a high-fat diet exhibited markedly increased myocardial levels of inflammatory adipokines, including adiponectin and fibroblast growth factor-1 (FGF-1). In addition, these animals developed significant electrocardiographic abnormalities and left ventricular remodeling associated with reduced ejection fraction. In a similar approach, Sommariva and colleagues [29] fed Pkp2-mutant mice a high-fat diet for three months to test the hypothesis that elevated cholesterol levels and oxidative stress would enhance cardiac lipid accumulation. Compared with wild-type controls, mutant mice displayed more extensive fatty infiltration on histological analysis, increased oxidative stress, and higher expression of PPARγ and CD36. High-fat diet exposure also induced right ventricular dysfunction at echocardiography. Importantly, the addition of lipid-lowering therapy (atorvastatin 20 mg/kg) to the high-fat diet resulted in reduced myocardial lipid accumulation, a marked decrease in PPARγ expression, and improvement in echocardiographic parameters of right ventricular size and function [29].

Lin et al. [56] generated an ACM mouse model through cardiac-specific DSG2 knockout, reproducing a phenotype closely resembling human ACM. DSG2-deficient mice showed the downregulation of adipose triglyceride lipase, fatty acid transport proteins, and enzymes involved in fatty acid β-oxidation. This impairment in fatty acid oxidation was linked to the inhibition of the mechanistic target of rapamycin (mTOR)/4EBP1/PPARα signaling pathway. Notably, the ACM phenotype could be reversed by PPARα activation using either fibrates or an adeno-associated virus serotype 9-mediated (AAV9) PPARα gene delivery approach. As a functional counterpart of PPARγ in lipid metabolism regulation, PPARα thus emerges as a potential target for disease-modifying therapies [56].

While these *in vivo* findings are promising, it is critical to note that animal models may not fully recapitulate the complexity of human ACM; therefore, these results provide a rationale for clinical investigation rather than a definitive proof of therapeutic efficacy.

### 3.3 Humans’ Data on the Horizon

To date, no randomized clinical trials have evaluated the impact of lipid-lowering therapies in patients with ACM. Nevertheless, several observational studies have investigated the relationship between lipid metabolism dysregulation and clinical outcomes in this population.

ACM is characterized by profound lipid metabolism dysregulation. Acylation-stimulating protein (ASP) is a molecule generated through the alternative complement pathway that regulates lipogenesis and triglyceride storage [57]. ASP’s role as a lipogenic adipokine that promotes triglyceride synthesis and storage represents a complex metabolic pivot point; it can be interpreted as a biomarker of systemic metabolic dysfunction that parallels underlying cardiac lipid abnormalities, or as an active contributor to disease progression through its influence on inflammatory pathways and metabolic stress [38,58]. Given that ASP is a byproduct of the significant complement activation seen in ACM, elevated levels may reflect a combination of ongoing myocardial inflammation, complement-mediated tissue injury and fibro-fatty replacement, and a sustained chronic inflammatory state that drives progressive ventricular remodeling and heart failure [38,58,59]. Ren et al. [60] enrolled 111 ACM patients and 106 healthy controls and measured plasma levels of ASP. Plasma ASP concentrations were significantly higher in ACM patients, with a similar pattern observed in myocardial explant samples. Elevated ASP levels were associated with a significantly higher incidence of heart failure events [60]. Statins have been hypothesized as a multi-tool in ACM management, providing potent anti-inflammatory effects to suppress cytokines [61,62], while simultaneously modulating lipid metabolism to improve the overall metabolic milieu [63]. Beyond these core actions, their potential to slow fibro-fatty remodeling and rebalance adipokines [64,65]—though the latter remains a complex metabolic balancing act—suggests a theoretical ability to stall disease progression on several systemic and local fronts, while the actual clinical impact of these pleiotropic effects in ACM patients has not yet been demonstrated.

Sommariva et al. [29] assessed plasma concentrations of oxLDL and 13-HODE in ACM patients and healthy controls, demonstrating significantly higher levels of both biomarkers in the ACM cohort. A difference in mean oxLDL levels was also observed between patients with overt ACM and mutation-positive but clinically unaffected relatives, independently of the underlying gene variant. Furthermore, increased plasma oxLDL and 13-HODE levels were mirrored by enhanced oxidative stress at the myocardial tissue level. In a subset of patients undergoing cardiac magnetic resonance imaging, oxLDL concentrations above a cutoff of 86 ng/mL were associated with a higher prevalence of right ventricular dysfunction, biventricular dysfunction, and right ventricular wall motion abnormalities. Kaplan–Meier analysis showed that survival free from major arrhythmic events over a 5-year follow-up was significantly higher among ACM patients with lower oxLDL levels [29].

While elevated oxLDL correlates with disease severity, a significant limitation of these observational findings is the risk of reverse causality. It remains plausible that metabolic dysfunction is a secondary consequence of myocardial structural failure rather than a primary driver. Furthermore, these studies are often small and retrospective, introducing potential selection bias that may skew the perceived strength of the metabolic-phenotype association.

## 4. Lipid-Lowering Therapy as a Candidate Disease-Modifying Approach

Beyond their established role in modulating the mevalonate pathway to suppress hepatic cholesterol synthesis [66], statins exert diverse pleiotropic effects that are pivotal in myocardial and vascular remodeling [66]. These non-lipid-lowering properties encompass the attenuation of systemic inflammation, the restoration of endothelial nitric oxide bioavailability, and the enhancement of plaque stability [66,67,68]. Additionally, statins demonstrate a robust capacity to inhibit vascular smooth muscle cell dysregulation and platelet-mediated thrombotic cascades [67]. These effects operate via discrete molecular axes—most notably the isoprenylation of small GTP-binding proteins—thereby rendering their clinical efficacy independent of serum LDL-cholesterol reduction [67].

### 4.1 Reimagining Statins: Decoupling Lipid Lowering From Myocardial Stabilization

The pharmacological landscape of 3-hydroxy-3-methylglutaryl-coenzyme A (HMG-CoA) reductase inhibitors encompasses multiple generations of compounds, ranging from first-generation natural statins (lovastatin, pravastatin, and simvastatin) to subsequent synthetic analogs (fluvastatin, atorvastatin, and rosuvastatin) [66]. These agents differ significantly in their lipophilicity, potency, and the magnitude of pleiotropic signaling induction. While the cardiovascular benefits of statins are well-established in atherosclerotic disease, clinical interest in their application for non-ischemic cardiomyopathies has grown. Specifically, large-scale observational data and randomized subsets have demonstrated that atorvastatin and simvastatin are associated with significant mortality reductions in patients with non-ischemic dilated cardiomyopathy (NIDCM) [69]. The literature provides extensive evidence regarding the efficacy of statins in reducing mortality and arrhythmic events among patients with NIDCM. In a sub-analysis of 458 patients from the DEFINITE study conducted by Goldberger et al. [70], the authors concluded that statin therapy is associated with a profound reduction in all-cause mortality and sudden cardiac death. This therapeutic advantage is hypothesized to originate from the pleiotropic effects of statins. A comprehensive review by Verma and Figueredo [71] positioned statins as a pathophysiological intervention capable of modifying the disease substrate in NIDCM, advocating for their use as a complementary therapy to stabilize myocardial electrical activity and arrest the progression of ventricular dysfunction. In the randomized controlled trial conducted by Bielecka-Dabrowa et al. [72] regarding DCM, the supplementation of optimal heart failure therapy with atorvastatin was shown to attenuate inflammation, enhance cardiac function, and significantly improve long-term survival outcomes. Similar results were observed in the BEST trial analysis, where statin use was strongly associated with better survival in patients with advanced nonischaemic dilated cardiomyopathy [73]. While the benefits observed in NIDCM suggest that specific statins exert a protective effect on the myocardial substrate beyond coronary event prevention [71,74], applying these findings to ACM remains strictly hypothesis-generating. Given the distinct fibro-fatty pathophysiology of ACM compared to the predominantly fibrotic remodeling in NIDCM, these implications require dedicated clinical validation rather than being viewed as evidence-confirming. This inconsistency in the broader heart failure literature highlights the risk of assuming a definitive benefit in the specific, and pathophysiologically distinct, context of ACM.

Extensive research underscores the anti-arrhythmic efficacy of statin therapy across a diverse spectrum of cardiac pathologies. Notably, a meta-analysis by Wanahita et al. [75] demonstrated a 31% reduction in the incidence of ventricular tachyarrhythmias. Furthermore, Buber et al. [76] reported a 77% decrease in life-threatening arrhythmic events (fast ventricular tachycardia (VT)/ventricular fibrillation (VF) or death) specifically within the non-ischemic cardiomyopathy population enrolled in the MADIT-CRT trial. Similarly, data derived from the MADIT-II trial demonstrated a significant anti-arrhythmic benefit associated with statin therapy in patients with ICDs. Statin administration correlated with a reduced risk of both VT/VF and overall cardiac mortality, further supporting their role in stabilizing the electrical substrate [77]. A summary of statin therapy in patients with NIDCM is shown in Table 1 (Ref. [69,70,72,73,74,75,76]).

**Table 1.** **Summary of studies evaluating statin therapy in patients with non-ischemic dilated cardiomyopathy or broader cardiomyopathy populations**.

Study/Trial	Population	Statin (dose/info)	Outcomes
DEFINITE Trial post-hoc analysis [70]	458 NIDCM patients	Various statins were used at baseline (predominantly atorvastatin/simvastatin)	Statin therapy associated with 78% mortality reduction (HR 0.22, 95% CI 0.09–0.55, *p* = 0.001); marked reduction in arrhythmic sudden death; trend toward fewer appropriate ICD shocks
SCD-HeFT analysis [74]	2521 patients: ischemic + non-ischemic cardiomyopathy (LVEF ≤35%, NYHA II–III)	Various statins	Statin use is associated with lower all-cause mortality overall (HR 0.70, 95% CI 0.58–0.83, *p* = 0.001); benefit is similar in non-ischemic cardiomyopathy (HR 0.67, 95% CI 0.47–0.96) and in ICD and non-ICD subgroups
MADIT-CRT subgroup [76]	821 NIDCM patients with CRT-D	Various statins (type/dose not specified)	Time-dependent statin therapy associated with 77% reduction in fast VT/VF or death (*p* < 0.001) and 46% fewer appropriate ICD shocks (*p* = 0.01)
BEST trial subgroup post-hoc analysis [73]	1024 NIDCM patients (NYHA III–IV, LVEF ≤35%)	Any statin at enrollment (type not specified)	Statin use is independently associated with lower all-cause mortality (HR 0.38, 95% CI 0.18–0.82, *p* = 0.0134) and cardiovascular death (HR 0.42, 95% CI 0.18–0.95, *p* = 0.037) after multivariable adjustment
Zhonghua Yi Xue Za Zhi Single-center retrospective cohort [69]	315 NIDCM patients (median follow-up 45.1 months)	Early atorvastatin or simvastatin vs no statin	All-cause mortality 17.2% with statin vs 37.4% without (*p* = 0.003); in NYHA III–IV, RR of death with statin 0.25 (95% CI 0.081–0.778, *p* = 0.017); benefit independent of LDL-C and background ACEI/β-blocker use
Atorvastatin DCM RCT prospective randomized trial [72]	68 DCM patients (LVEF ≤40%, 5-year follow-up)	Atorvastatin 40 mg for 2 months, then 10–20 mg/day vs no statin	Statin group: lower IL-6, TNF-α; reduced LV diameters; LVEF improved from 32.0 ± 6.4% to 38.8 ± 8.8% (*p* = 0.016); 5-year survival significantly higher with statin (log-rank *p* = 0.005)
Meta-analysis on 9 prospective studies [75]	150,953 patients (ischemic and NIDCM)	Various statin use	Statins associated with 31% relative risk reduction in VT/VF (pooled RR 0.69, 95% CI 0.58–0.83, Egger’s test *p* value = 0.957)

CRT-D, cardiac resynchronization therapy defibrillator; DCM, dilated cardiomyopathy; HR, hazard ratio; ICD, implantable cardioverter-defibrillator; IL-6, interleukin-6; LVEF, left ventricular ejection fraction; LDL-C, low-density lipoprotein cholesterol; LV, left ventricle; NIDCM, non-ischemic dilated cardiomyopathy; NYHA, New York Heart Association; RR, risk ratio; TNF-α, tumour necrosis factor-α; VF, ventricular fibrillation; VT, ventricular tachycardia; ACEI, angiotensin-converting enzyme inhibitors.

In the context of ACM, atorvastatin—characterized by its high lipophilicity and potent HMG-CoA reductase inhibition—has been preferentially selected in emerging trials due to its proven capacity to modulate myocardial remodeling and inflammatory signaling in other non-ischemic settings [78]. The transition from NIDCM evidence to ACM clinical protocols is predicated on the hypothesis that the anti-fibrotic and metabolic-stabilizing properties of high-potency statins may arrest the progressive fibro-fatty replacement of the ventricular myocardium. In the Pkp2 heterozygous knock-out murine model subjected to a high-fat diet challenge, atorvastatin successfully mitigated cardiac lipid deposition and right-ventricular impairment. These findings suggest that reducing oxidized lipid species, perhaps augmented by pleiotropic mechanisms, can suppress the ACM phenotype *in vivo* [29]. The therapeutic efficacy observed likely stems from a synergy between systemic LDL reduction and non-lipid-mediated pathways, including the attenuation of inflammatory responses, modulation of Wnt/β-catenin and Dickkopf protein (DKK) signaling cascades, suppression of sympathetic overactivity, and potential anti-arrhythmic effects [29,79,80,81].

While these findings preclinically suggest a disease-modifying role for statins for patients with desmosomal phenotypes characterized by elevated oxLDL, randomized clinical trials are necessary to determine if these effects translate to the human desmosomal phenotype. Considering robust clinical evidence demonstrating that intensive LDL-C reduction mitigates atherosclerotic cardiovascular events—independent of the pharmacological mechanism employed [79,82]—statin therapy may offer a dual therapeutic benefit for ACM patients, who often present with concomitant traditional cardiovascular risk factors. The ongoing SEARCH clinical trial is currently evaluating the efficacy of atorvastatin as a potential disease-modifying intervention in patients with ACM [78]. This investigation is predicated on preliminary evidence suggesting that statins may arrest disease progression by lowering systemic levels of oxLDL and modulating key pathogenic signaling pathways—such as those involved in adipogenesis and fibrosis—within the myocardium.

### 4.2 The Untapped Potential: Non‑Statin Lipid‑Lowering Agents

Although existing literature provides a comprehensive characterization of the cardiovascular implications of ezetimibe [83,84], there remains a paucity of data regarding its specific impact on the ACM phenotype. A notable finding by Sander et al. [85] suggests that ezetimibe may augment mitochondrial calcium uptake, thereby potentially suppressing cardiac arrhythmogenesis. However, these anti-arrhythmic properties have yet to be validated within the specific context of ACM-related myocardial remodeling.

Cumulative evidence indicates that PUFAs may significantly enhance cardiac performance in patients with non-ischemic cardiomyopathy. Specifically, Nodari et al. [86] demonstrated that these fatty acids exert favourable effects on arrhythmic risk profiles in patients diagnosed with idiopathic dilated cardiomyopathy. In a subsequent 2011 study [87], the authors established that n-3 PUFAs supplementation yielded a 10.4 ± 9.5% absolute increase in left ventricular ejection fraction, accompanied by a significant enhancement in functional capacity among paucisymptomatic cohorts. From a mechanistic perspective, PUFAs appear to exert cardioprotective effects by stabilizing myocardial electrical activity through the modulation of sodium and calcium ion channels, thereby mitigating arrhythmic risk [88,89]. Furthermore, Dimitrow and Jawien [90] observed that these fatty acids provide potent anti-arrhythmic benefits by stabilizing cardiomyocyte membranes and inhibiting pro-arrhythmogenic ion currents. Notwithstanding these promising preliminary findings, large-scale prospective trials are requisite to definitively establish the clinical efficacy of PUFAs in patients affected by ACM.

The majority of clinical data concerning fibrates, proprotein convertase subtilisin/kexin type 9 (PCSK9) inhibitors, bempedoic acid, and siRNA are derived from populations with atherosclerotic cardiovascular disease, rather than ACM cohorts [82,91,92,93,94,95,96]. While these agents significantly attenuate levels of LDL-C and triglyceride-rich lipoproteins—thereby reducing the incidence of major adverse cardiovascular events (MACE)—PCSK9 inhibitors also demonstrate the capacity to improve arterial stiffness and microvascular function. Such findings suggest pleiotropic vascular benefits that extend beyond their primary lipid-lowering mechanisms [82,96,97,98]. Also, PCSK9 inhibition reduced cardiac fibrosis in ischemia-reperfusion injury models by suppressing the transforming growth factor beta 1 (TGF-β1)/Smad3 pathway and inflammatory responses [99,100]. Although PCSK9 has been shown to have direct effects on cardiomyocytes beyond lipid metabolism—including promoting cardiomyocyte apoptosis, autophagy, and inflammation—currently there is no clinical evidence that PCSK9 inhibition improves outcomes specifically in ACM [99,101]. Furthermore, although PCSK9-targeted therapies and cholesteryl ester transfer protein (CETP) inhibitors are among the few classes capable of substantially reducing lipoprotein(a) levels, their specific impact on ACM-related clinical outcomes remains to be elucidated [95].

Bempedoic acid activates AMP-activated protein kinase (AMPK) signaling, leading to anti-inflammatory, antioxidant, and anti-fibrotic effects [102]. In hypertensive rat models, it attenuated cardiac remodeling by inhibiting NF-κB/TGF-β pathways, reducing endoplasmic reticulum stress, and preventing cardiac fibrosis [103,104]. Given that inflammation and fibro-fatty remodeling are central to the pathophysiology of ACM, and that bempedoic acid may exert anti-inflammatory and anti-fibrotic properties in cardiac tissue, there might be a theoretical rationale for investigating its potential as a therapeutic strategy [105]. However, in the absence of even preclinical animal data, such a strategy remains entirely speculative.

**Fig. 2.**
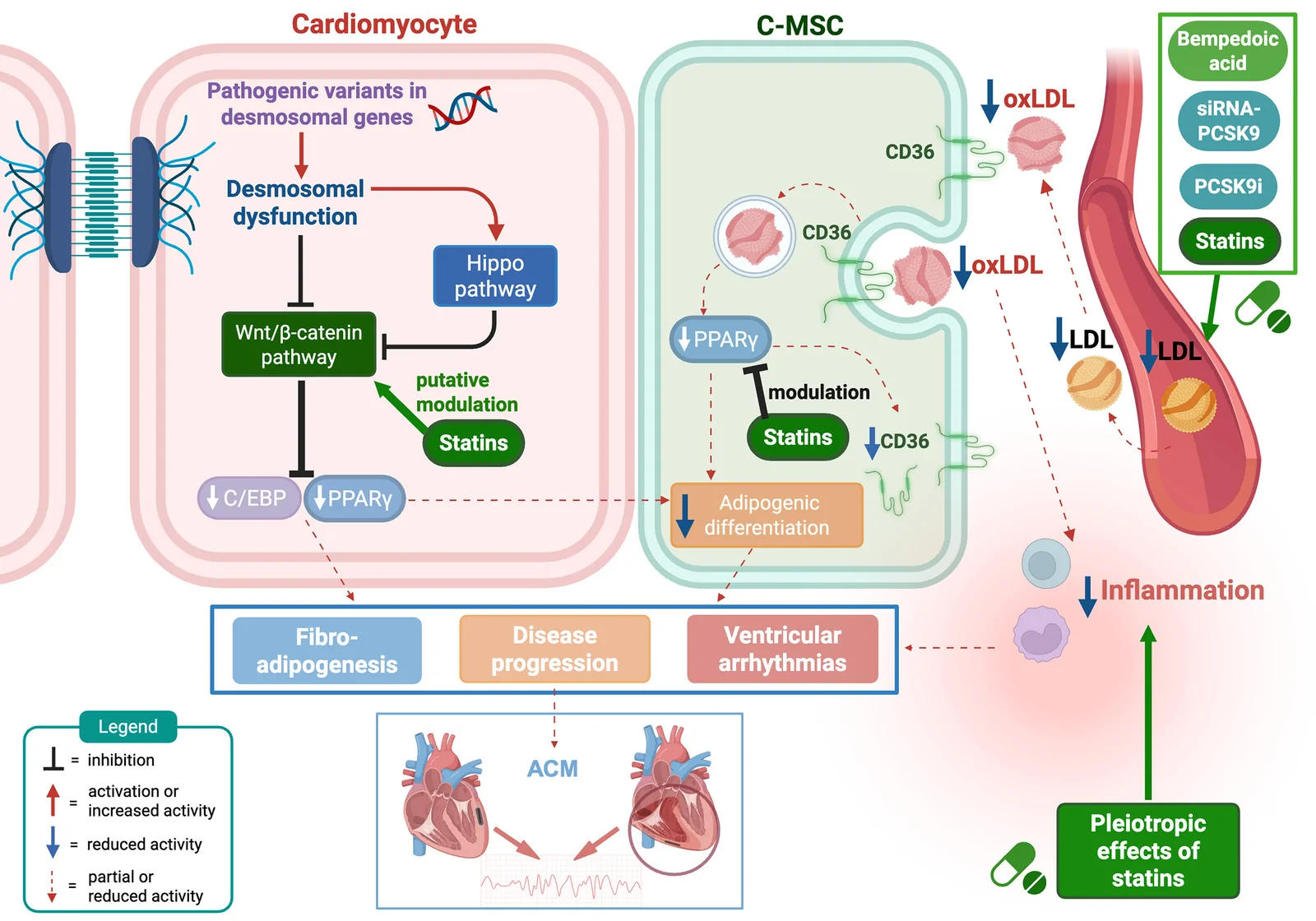
**From desmosomal dysfunction to potential metabolic targeting in arrhythmogenic cardiomyopathy**. The figure provides an integrated schematic overview of the molecular mechanisms underlying arrhythmogenic cardiomyopathy and the putative modulatory effects of lipid-lowering therapies. ACM, arrhythmogenic cardiomyopathy; CD36, cluster of differentiation 36; C/EBP, CCAAT/enhancer-binding protein; C-MSC, cardiac mesenchymal stromal cell; LDL, low-density lipoprotein; oxLDL, oxidized low-density lipoprotein; PCSK9, proprotein convertase subtilisin/kexin type 9; PCSK9i, proprotein convertase subtilisin/kexin type 9 inhibitors; PPARγ, peroxisome proliferator activated receptor gamma; siRNA, small interfering ribonucleic acid. This figure was created with BioRender.com.

Regarding siRNA therapies, their primary action is to provide a potent and sustained reduction in circulating LDL-C levels by inhibiting the hepatic synthesis of PCSK9 in hepatocytes. While this could theoretically limit the substrate available for oxLDL formation [29], it is important to note that these agents act purely on the quantitative levels of LDL-C. Unlike statins, which have well-documented pleiotropic effects—including direct anti-inflammatory and antioxidant properties that have shown benefit in ACM animal models—siRNAs do not currently offer a known additional mechanistic advantage for modulating the specific myocardial lipotoxicity seen in ACM. The metabolic targets of the lipid-lowering therapy are shown in Fig. 2.

## 5. Gene Therapies

The integration of gene therapy into the ACM treatment landscape offers a definitive solution to the metabolic dysfunction driving disease progression. While pharmacological interventions like statins aim to mitigate the downstream effects of the oxLDL/CD36/PPARγ axis, gene therapy addresses the root cause by restoring the structural and signaling integrity of the desmosome. By delivering functional copies of mutated genes—such as *PKP2* or *DSC2*—this approach re-establishes the correct protein architecture at the intercalated disc. Restoring these primary structural components effectively “deactivates” the pathological molecular switch; with desmosomal stability regained, the intracellular signaling cues that erroneously divert progenitor cells toward an adipogenic, fibro-fatty lineage are silenced. Consequently, gene therapy does not merely manage symptoms but fundamentally recalibrates the myocardial environment, arresting the lipid-driven replacement of cardiac tissue at its genetic source [106,107].

Gene therapy for ACM represents a burgeoning therapeutic frontier, with adeno-associated virus (AAV)-mediated gene replacement targeting *PKP2* mutations currently spearheading clinical development. As the most advanced strategy to date, *PKP2* replacement therapy has demonstrated significant translational potential; notably, the investigational agent LX2020 (an AAV-vector carrying the *PKP2* transgene) recently received Food and Drug Administration (FDA) clearance for clinical evaluation. This milestone follows robust preclinical data in *PKP2* mutant murine models, which showed dose-dependent improvements in survival rates, a reduction in ectopy, and the functional amelioration of both left and right ventricular ejection fractions [108,109]. Parallel to viral-mediated approaches, modified mRNA therapy has emerged as a promising alternative in preclinical settings. Delivery of *DSC2* mRNA, utilized both with and without lipid nanoparticle encapsulation, has successfully normalized cardiac morphology and systolic function in DSC2-deficient models [110]. Gene replacement therapy for *DSP* is in early preclinical development, with approaches like *PKP2* being explored using AAV-mediated delivery. Mutation-agnostic connexin-43 (Cx43) restoration has shown efficacy in DSP-deficient ACM models [111]. Pharmacological pathway modulation targeting Wnt/β-catenin signaling has also shown promise in zebrafish models of *DSP* deficiency, where both genetic and pharmacological rescue of Wnt signaling reversed the ACM phenotype [112]. Modified mRNA therapy represents a novel approach for *DSG2* deficiency. AAV-mediated gene replacement has demonstrated proof-of-concept in patient cardiomyocytes with complete *DSG2* deficiency, where AAV-DSG2 delivery significantly recovered contractile force in three-dimensional tissue constructs [113]. Mutation-agnostic Cx43 restoration also showed efficacy in DSG2-mutant human cardiomyocytes, alleviating physiological deficits [111]. Gene replacement therapy for *JUP* has shown proof-of-concept in Naxos disease models. Normalization of plakoglobin levels to wild-type restored cardiac function in mice with the *JUP2157del2* mutation, demonstrating that the disease results from haploinsufficiency rather than gain-of-function [114]. This finding supports the feasibility of AAV-mediated *JUP* gene replacement. AAV-mediated gene replacement therapy has shown efficacy in preclinical models of recessive desminopathies. In desmin knockout mice, AAV9-mediated wild-type desmin transfer partially reconstituted desmin protein expression, restored the extrasarcomeric desmin-syncoilin network, reduced cardiac fibrosis and hypertrophy, and improved systolic left ventricular function [115,116]. For rarer ACM genes, including transmembrane protein 43 (*TMEM43*), lamin A/C (*LMNA*), RNA-binding motif protein 20 (*RBM20*), titin (*TTN*) and phospholamban (*PLN*), gene therapy development remains in early conceptual or preclinical stages. General approaches being considered include AAV-mediated gene replacement for haploinsufficiency mutations, clustered regularly interspaced short palindromic repeats (CRISPR)-based editing for specific variants, and suppression-replacement strategies for dominant-negative mutations [106,117]. These advancements underscore a pivotal transition toward precision molecular interventions aimed at correcting the underlying genetic drivers of the ACM phenotype. A summary of the genes involved, along with their underlying mechanisms and potential therapeutic strategies currently in development, is presented in Table 2 (Ref. [108,109,110,111,113,114,115,116,117,118]).

**Table 2.** **Summary of the genes, underlying mechanisms and potential therapeutic strategies**.

Gene	Protein (location)	Frequency	Mechanism of disease	Gene therapy strategies	References
*PKP2*	Plakophilin-2 (desmosome)	20–45% (most common)	Desmosomal dysfunction Wnt/β-catenin suppression → adipogenic/fibrotic switch	AAV-mediated gene replacement	[108,109]
*DSP*	Desmoplakin (desmosome)	2–15% (most common in ALVC)	Desmosomal structural compromise Wnt pathway suppression	Gene replacement therapy (preclinical) Potential for AAV-mediated gene therapy	[111]
*DSG2*	Desmoglein-2 (desmosome)	4–15%	Transmembrane desmosomal protein dysfunction Nuclear PPARγ accumulation → oxidative stress	Modified mRNA therapy PPARγ antagonism	[113,118]
*DSC2*	Desmocollin-2 (desmosome)	2–7%	Transmembrane desmosomal protein loss → intercellular junction failure	Modified mRNA therapy Potential AAV-mediated gene replacement	[110]
*JUP*	Plakoglobin (desmosome)	1% (except Naxos disease)	Wnt/β-catenin and Hippo pathway suppression	Gene replacement therapy (preclinical)	[114]
*DES*	Desmin (cytoskeleton)	1–2%	Impaired cytoskeletal integrity	Gene replacement or editing (preclinical)	[115,116]
*TMEM43*	Transmembrane protein 43 (nuclear envelope)	1% (founder effect in Newfoundland)	Nuclear envelope protein dysfunction Wnt pathway suppression	Gene replacement or silencing strategies (preclinical)	[117]
*PLN*	Phospholamban (sarcoplasmic reticulum)	1%	Ca^2+^ handling dysfunction *p.Arg14del* founder mutation → protein aggregation, lipotoxicity;	Antisense oligonucleotide therapy Allele-specific silencing for founder mutation	[117]

AAV, adeno-associated virus; ALVC, arrhythmogenic left ventricular cardiomyopathy; *DES*, desmin; *DSC2*, desmocollin-2; *DSG2*, desmoglein-2; *DSP*, desmoplakin; *JUP*, plakoglobin; mRNA, messenger ribonucleic acid; PKP2, plakophilin-2; PLN, phospholamban; PPARγ, peroxisome proliferator-activated receptor gamma; *TMEM43*, transmembrane protein 43; Wnt/β-catenin, wingless-related integration site/beta-catenin signaling pathway.

## 6. What We Have Now…and What We Will Have

### 6.1 Current Guidelines

Current guidelines about cardiomyopathies propose a treatment strategy focused on the prevention of sudden cardiac death and on heart failure therapy; however, there are currently no strong indications for aetiology disease-modifying therapies for ACM. According to the European Society of Cardiology (ESC) guidelines, pharmacological recommendations include the initiation of beta-blockers at the maximum tolerated dose [119]. Amiodarone and flecainide should be considered in cases of inadequate control of ventricular arrhythmias. Catheter ablation of ventricular tachycardia, possibly with an epicardial approach guided by 3D electro-anatomical mapping, should be considered in patients with incessant ventricular tachycardia or receiving appropriate ICD interventions for ventricular tachycardia despite pharmacological therapy with beta-blockers [119]. Abstinence from sports activity is recommended due to the dose-dependent relationship with the severity of ACM. ICD implantation is recommended for secondary prevention, both in cases of cardiac arrest (Class I) and in cases of hemodynamically tolerated ventricular tachycardia (Class IIa) [119].

For primary prevention, the guidelines suggest an individualized evaluation based on the presence of high-risk features for arrhythmic events: arrhythmic syncope, non-sustained ventricular tachycardia, right ventricular ejection fraction <40%, left ventricular ejection fraction <45%, and inducible sustained monomorphic ventricular tachycardia during programmed ventricular stimulation [119]. Despite these robust recommendations for rhythm management, current guidelines offer limited guidance on the comprehensive management of non-arrhythmic disease aspects and the underlying structural progression of the cardiomyopathy.

### 6.2 Emerging Clinical Evidence and Future Directions

Growing evidence in the pathophysiology of ACM, together with advances in translational technologies, is creating new opportunities for tailored therapeutic strategies aimed at restoring the physiological metabolic balance of the myocardium. To date, *in vitro* and animal studies consistently support a pivotal role of lipid metabolism disruption in disease onset and progression. However, human data remain limited to small observational studies, and no randomized clinical trial has yet investigated whether lipid-lowering therapy can modify the natural history of the disease.

The SEARCH study [78] (Statins in Arrhythmogenic Right Ventricular Cardiomyopathy; NCT06922994) represents the first randomized, double-blind, placebo-controlled phase II clinical trial specifically powered to evaluate whether atorvastatin can attenuate the structural remodeling associated with ACM. By designating right ventricular function—quantified via right ventricle (RV) free-wall longitudinal strain—as its primary endpoint, the study utilizes this sensitive imaging metric to detect subtle changes in myocardial contractility that often precede global systolic decline [78]. This design reflects the hypothesis that metabolic modulation primarily influences the structural substrate of the disease before the onset of overt electrical instability; consequently, clinical “hard endpoints”, such as premature ventricular contraction burden, sustained and non-sustained ventricular arrhythmias, and the incidence of appropriate ICD interventions, are categorized as secondary endpoints. To further delineate the trial’s scope, morphological stability is assessed via echocardiography and cMRI, complemented by the longitudinal profiling of an expansive panel of circulating biomarkers. This panel integrates markers of myocardial injury and wall stress (B-type natriuretic peptide (BNP), N-terminal pro-B-type natriuretic peptide (NT-proBNP), troponin I, bridging integrator 1 (BIN1), heat-shock protein 70 (HSP70)) with lipidomic and oxidative stress signatures (LDL, oxLDL, 4-hydroxynonenal (4-HNE)), systemic inflammation (C-reactive protein (CRP), IL-6, TNF-α), and fibrotic turnover (tissue inhibitor of metalloproteinases-1 (TIMP1), procollagen 3 N-terminal peptide (PIIINP), suppression of tumorigenicity 2 (ST2), TGF-β, galectin-3 (Gal-3)) [8,78]. By synthesizing data from this multidimensional characterization, the trial aims to investigate the mechanistic role of lipid metabolism and oxidative stress in the phenotypic progression of ACM, investigating whether the pleiotropic effects of statins could decelerate myocardial substrate remodeling, mitigate electrical vulnerability, and redefine the therapeutic landscape for this patient population. A major limitation in validating these therapies is the inherent difficulty in powering clinical trials for rare diseases like ACM. The low prevalence of the disease and the slow progression of structural remodeling make it challenging to reach statistically significant endpoints within standard trial timeframes, which may result in underpowered studies that fail to detect subtle but meaningful clinical benefits.

Despite the promise of statins, a significant evidence gap remains regarding other lipid-modulating pathways. There is currently no clinical data exploring whether the profound LDL-lowering of PCSK9 inhibitors, bempedoic acid, siRNA-based therapies, or the targeted triglyceride reduction of fibrates can specifically influence intramyocardial lipid accumulation and fibro-fatty replacement. Future research must prioritize mechanistic studies to elucidate the exact molecular pathways through which these agents interact with the ACM substrate. Identifying these specific molecular targets—such as the modulation of PPAR signaling or the inhibition of intracellular lipid droplet formation—is essential to transition from empirical lipid-lowering to precision metabolic therapies tailored to the ACM-specific lipotoxic environment. As a potential future perspective, given the putative role of oxLDL in ACM pathophysiology, a plausible therapeutic target may be LOX-1, the key scavenger receptor for oxLDL, which represents a central pathogenic mechanism in cardiovascular disease. In this regard, a phase 2b study in the post-myocardial infarction setting—enrolling patients with myocardial infarction and residual inflammation (high-sensitivity C-reactive protein ≥1 mg/L)—evaluated MEDI6570, a monoclonal antibody acting as a LOX-1 antagonist [120]. Although the trial did not achieve statistical significance for the primary endpoint (change in non-calcified plaque volume in the most diseased coronary segment, assessed by coronary computed tomography angiography), exploratory analyses showed significant reductions in free soluble LOX-1 (sLOX-1) from baseline, alongside reductions in IL-6. Collectively, these findings suggest that pharmacological LOX-1 antagonism may represent a rational translational avenue to explore in ACM, potentially addressing both oxLDL-driven signaling and the inflammatory milieu that contributes to disease progression.

Further studies are warranted to clarify whether targeting metabolic pathways can effectively stabilize, or potentially slow, the progression of ACM. If confirmed, this strategy could also open the possibility of preventive interventions in genotype-positive/phenotype-negative individuals to delay or prevent phenotypic expression. In this context, metabolomics (and more specifically lipidomics) may represent a key translational bridge between experimental evidence and clinical application in ACM. Given the central role of lipid handling abnormalities in preclinical models, comprehensive metabolic profiling could help define disease-specific lipid signatures associated with early myocardial remodeling. Such an approach may allow the identification of subtle metabolic alterations in genotype-positive/phenotype-negative individuals, potentially preceding detectable structural changes on cardiac imaging. Beyond pathophysiological insights, metabolomic profiling could provide circulating biomarkers useful for risk stratification and for monitoring response to metabolism-modulating therapies, including lipid-lowering agents. In the context of interventional trials, integrating lipidomic data with cardiac magnetic resonance parameters and arrhythmic burden may help clarify whether metabolic modulation translates into measurable structural stabilization. Ultimately, combining genetic, imaging, and metabolomic information could support a precision medicine framework, identifying patient subgroups most likely to benefit from targeted metabolic interventions and refining disease-modifying strategies in ACM. Patient selection could prioritize individuals with a high metabolic burden, specifically those with oxLDL levels exceeding 86 ng/mL, a threshold independently linked to increased ventricular fat and arrhythmic risk [29]. Furthermore, timing is critical; clinical integration is likely most effective when initiated during the preclinical ‘concealed’ phase or the ‘hot phases’ of the disease, where the pleiotropic stabilization of the intercalated disc can occur before irreversible fibro-fatty replacement. For the clinician, this involves a transition from standard lipid monitoring to a targeted molecular strategy that uses statins as an adjunct to—rather than a replacement for—traditional β-blocker therapy. The introduction of statins into standard ACM care must be weighed against the risks of long-term statin therapy in a relatively young patient population, including potential side effects like myalgia or metabolic changes. The absence of longitudinal safety data specific to ACM patients represents a critical gap that must be addressed before a widespread shift in clinical practice can be recommended.

## 7. Conclusions

The current clinical management of ACM is constrained by a reactive “damage control” paradigm, which prioritizes the secondary prevention of SCD and the mitigation of arrhythmic burden. This approach creates a significant therapeutic void by addressing the terminal electrical manifestations of the disorder while neglecting the upstream oxLDL/CD36/PPARγ metabolic axis that orchestrates myocardial decay. By failing to modulate this “molecular switch”, contemporary strategies offer no resistance to the aberrant differentiation of resident progenitor cells into adipogenic lineages, ultimately permitting the irreversible fibro-fatty replacement of functional myocardium.

To bridge this gap, research is increasingly exploring a shift toward substrate stabilization, with the aim of investigating whether pathogenic progression can be arrested prior to the onset of irreversible structural deterioration. Resolving this clinical deficit requires the integration of lipidomic profiling to identify metabolic triggers at a sub-clinical stage, thereby closing the diagnostic gap between genetic susceptibility and phenotypic expression. Furthermore, the potential of high-potency statins warrants re-evaluation as a targeted intervention to stabilize the intercalated disc and neutralize pro-adipogenic lipid species. Potential future strategies might include targeting patients with a high metabolic burden and initiating therapy during ‘concealed’ or ‘hot phases’. Furthermore, these agents could be integrated as a synergistic adjunct to standard β-blockers to potentially stabilize the myocardial substrate. Establishing this metabolic interventional model depends on high-powered longitudinal evidence, such as the SEARCH study, to transition the field from palliative symptom management toward proactive, disease-modifying therapies that preserve myocardial identity.

## Abbreviations

AAV, adeno-associated virus; ACM, arrhythmogenic cardiomyopathy; AM, adipogenic medium; AMPK, AMP-activated protein kinase; ARVC, arrhythmogenic right ventricular cardiomyopathy; ASP, acylation-stimulating protein; BIN1, bridging integrator 1; BNP, B-type natriuretic peptide; CD36, cluster of differentiation 36; C/EBP, CCAAT-enhancer binding proteins; CETP, cholesteryl ester transfer protein; C-MSCs, cardiac mesenchymal stromal cells; cMRI, cardiac magnetic resonance; CRP, C-reactive protein; CStCs, cardiac stromal cells; Cx43, connexin-43; CRISPR, clustered regularly interspaced short palindromic repeats; DCM, dilated cardiomyopathy; DSC2, desmocollin-2; DSG2, desmoglein-2; DSP, desmoplakin; FABP4, fatty-acid binding protein 4; FASN, fatty-acid synthase; FGF-1, fibroblast growth factor-1; Gal-3, galectin-3; GCN5, general control non-depressible 5; HMG-CoA, 3-hydroxy-3-methylglutaryl-coenzyme A; HSP70, heat-shock protein 70; IL-1, interleukin-1; IL-6, interleukin-6; JUP, plakoglobin; LDL, low-density lipoprotein; LMNA, lamin A/C; LOX-1, lectin-like oxidized LDL receptor-1; LRPPRC, leucine-rich PPR motif-containing protein; MACE, major adverse cardiovascular events; mTOR, mechanistic target of rapamycin; NF-κB, nuclear factor kappa-light-chain-enhancer of activated B cells; NF2/Merlin, neurofibromatosis type 2/moesin-ezrin-radixin-like protein; NIDCM, non-ischemic dilated cardiomyopathy; NT-proBNP, N-terminal pro-B-type natriuretic peptide; oxLDL, oxidized low-density lipoprotein; SCD, sudden cardiac death; sPLA2, secretory phospholipase A2; ST2, suppression of tumorigenicity 2; PIIINP, procollagen 3 N-terminal peptide; PKC, protein kinase C; PKP2, plakophilin-2; PLN, phospholamban; PPARα, peroxisome proliferator-activated receptor alpha; PPARγ, peroxisome proliferator-activated receptor gamma; PUFA, polyunsaturated fatty acid; RBM20, RNA-binding motif protein 20; ROS, reactive oxygen species; siRNA, small interfering ribonucleic acid; SREBPs, sterol regulatory element-binding proteins; TAZ, transcriptional coactivator with PDZ-binding motif; TGF-β, transforming growth factor beta; TIMP1, tissue inhibitor of metalloproteinases-1; TMEM43, transmembrane protein 43; TNF-α, tumour necrosis factor alpha; TTN, titin; YAP, YES-associated protein; VF, ventricular fibrillation; VT, ventricular tachycardia; 13-HODE, 13-hydroxyoctadecadienoic acid; 15-LO, 15-lipoxygenase; 4-HNE, 4-hydroxynonenal.
